# Tackling the economic burden of postsurgical complications: would perioperative goal-directed fluid therapy help?

**DOI:** 10.1186/s13054-014-0566-1

**Published:** 2014-10-11

**Authors:** Gerard R Manecke, Angela Asemota, Frederic Michard

**Affiliations:** University of California San Diego, UCSD Medical Center, 200 West Arbor Drive, San Diego, CA 92103 USA; University of California San Diego School of Medicine, 200 West Arbor Drive, San Diego, CA 92103 USA; Global Medical Strategy, Critical Care, Edwards Lifesciences, Irvine, CA USA

## Abstract

**Introduction:**

Pay-for-performance programs and economic constraints call for solutions to improve the quality of health care without increasing costs. Many studies have shown decreased morbidity in major surgery when perioperative goal directed fluid therapy (GDFT) is used. We assessed the clinical and economic burden of postsurgical complications in the University HealthSystem Consortium (UHC) in order to predict potential savings with GDFT.

**Methods:**

Data from adults who had a major surgical procedure in 2011 were screened in the UHC database. Thirteen post-surgical complications were tabulated. In-hospital mortality, hospital length of stay and costs from patients with and without complications were compared. The risk ratios reported by the most recent meta-analysis were used to estimate the potential reduction in post-surgical morbidity with GDFT. Potential cost-savings were calculated from the actual and anticipated morbidity rates.

**Results:**

A total of 75,140 patients met the search criteria, and 8,421 patients developed one or more post-surgical complications (morbidity rate 11.2%). In patients with and without complications, in-hospital mortality was 12.4% and 1.4% (*P* <0.001), mean hospital length of stay was 20.5 ± 20.1 days and 8.1 ± 7.1 days (*P* <0.001) and mean direct costs were $47,284 ± 49,170 and $17,408 ± 15,612 (*P* <0.001), respectively. With GDFT, morbidity rate was projected to decrease to 8.0 - 9.3%, yielding gross costs savings of $43 M - $73 M for the study population or $569 - $970 per patient.

**Conclusion:**

Postsurgical complications have a dramatic impact (+172%) on costs. Potential costs savings resulting from GDFT are substantial. Perioperative GDFT may be recommended not only to improve quality of care but also to decrease costs.

## Introduction

Pay-for-performance programs and economic constraints call for solutions improving the quality of health care without increasing costs [1]. In this respect, the American Society of Anesthesiology is developing a perioperative surgical home program in order to optimize quality and continuity of care for surgical patients [2,3]. Perioperative goal directed fluid therapy (GDFT) is a general term referring to targeted hemodynamic and fluid management using parameters such as stroke volume, cardiac output, and/or oxygen delivery, in conjunction with standard vital signs in managing patients during and immediately after surgery. Many studies have shown decreased morbidity in major surgery when GDFT is used [4,5] and it is now recommended by the National Health Service in the UK [6], by the French Society of Anesthesiology [7], and by the Enhanced Recovery After Surgery (ERAS) society in Europe [8]. Despite this, adoption of GDFT in the United States is poor [9,10]. Perioperative GDFT may require the use of hemodynamic monitoring equipment, such as minimally invasive cardiac output monitoring, beyond that used for simple, routine surgery. The cost of monitoring equipment for GDFT may be a barrier to adoption.

By reducing complication rates, GDFT would be expected to decrease cost [11]. Indeed, a favorable financial impact of GDFT resulting from reduction of complication rates has been reported [12,13]. These studies, however, are not current, and involved the use of the pulmonary artery catheter for monitoring. With the exception of cardiac surgery and liver transplantation, the pulmonary artery catheter is no longer commonly used for perioperative hemodynamic optimization, having been replaced by less invasive technologies. To date there have been no reports of large, randomized, controlled trials assessing the financial impact of GDFT.

The University HealthSystem Consortium (UHC) is an alliance of 120 academic medical centers and 300 of their affiliated hospitals [14]. Query of their database allows the determination of complications and costs of care in surgical patients. These data, in conjunction with the outcome impact of GDFT that has been reported in the medical literature, allows an estimation of potential GDFT-related cost-savings in major surgery in the UHC. The goal of our study was twofold: to describe the clinical and economic burden of postsurgical complications in the UHC, and to predict the economic impact of GDFT implementation.

## Materials and methods

De-identified data from all adults who had major non-cardiac surgery in 2011 were screened in the UHC database. Permission to perform and report the results of this study was provided by the University of California San Diego Human Research Protections Program. This committee waived the need for informed consent, since this was a database study.

### Patient selection

Ten major surgical procedures were selected based on previous studies showing GDFT-associated positive outcomes [15-29]. Corresponding ICD9 codes were used to search specific procedures in the UHC database (Table 1). Because GDFT has thus far been shown to be effective only in adults, a restriction was used ensuring that only adult (≥18 years old) patients were queried.

**Table 1:** **Ten major surgical procedures queried in the UHC database, corresponding ICD9 codes, and studies showing morbidity reduction with GDFT**

**Surgical procedure**	**ICD9 codes**	**Author (reference)**
Abdominal aortic aneurysm open repair	38.44	Benes [15], Kuper [19], Lobo [20], Pearse [22], Wilson [29]
Aorto-iliac and peripheral bypass	39.25, 39.29	Bisgaard [16], Kuper [19], Pearse [22], Wilson [29]
Esophagectomy	42.40, 42.41, 42.42	Boyd [17], Kuper [19], Lobo [20], Pearse [22], Wilson [29]
Gastrectomy	43.5, 43.6, 43.7, 43.81, 43.89, 43.91, 43.99	Boyd [17], Kuper [19], Lobo [20], Pearse [22], Wilson [29]
Colectomy	45.71-45.76, 45.79, 45.81-45.83	Benes [15], Gan [18], Kuper [19], Noblett [21], Pearse [22], Ramsingh [24], Wakeling [28]
Resection of rectum	48.40, 48.43, 48.49-48-52, 48.59, 48.61-48.65, 48.69	Benes [15], Gan [18], Kuper [19], Noblett [21], Pearse [22], Ramsingh [24], Wakeling [28], Wilson [29]
Hepatectomy	50.22, 50.3	Pearse [22], Ueno [26],
Pancreatectomy	52.51-52.53, 52.59, 52.6, 52.7	Benes [15], Lobo [20], Ramsingh [24]
Total cystectomy	57.71, 57.79	Boyd [17], Gan [18], Kuper [19], Pearse [22], Pillai [23], Ramsingh [24], Wilson [29]
Femur & hip fracture repair	79.15, 79.25, 79.35, 79.85, 79.95	Kuper [19], Sinclair [25], Venn [27]

UHC, University HealthSystem Consortium; GDFT, goal directed fluid therapy.

### Clinical data collection

In-hospital postoperative complications queried, as defined by the UHC, included postoperative stroke, gastrointestinal hemorrhage, catheter-associated urinary tract infection, reopening of surgical site, acute myocardial infarction, coma or stupor, nosocomial pneumonia, wound infection, sepsis, pulmonary embolism or deep venous thrombosis, respiratory failure, hematoma and wound dehiscence. Morbidity rate was defined as the proportion of patients developing at least one complication during their hospital stay. Patients were sorted into two groups: those with complications and those without. For each group, in-hospital mortality and hospital length of stay (mean ± SD) were extracted from the UHC database and compared.

### Cost data collection and cost-savings projection

Direct costs (mean ± SD) related to the in-hospital treatment of patients with and without complications were obtained from the UHC database and compared. Direct costs are those associated with the actual procedures, they do not include overheads and wages for healthcare personnel. The most recent GDFT meta-analysis was used to estimate the potential reduction of postoperative morbidity from GDFT [5]. This meta-analysis reported an average odds ratio of 0.77 with a confidence interval ranging from 0.71 to 0.83. Potential cost-savings were determined by using the projected number of patients developing one or more complication and the estimated related costs. This analysis was performed for the entire cohort, as well as for each surgical procedure. The analysis assumes complete, new implementation of GDFT.

### Statistical analysis

In-hospital mortality, hospital length of stay and costs were compared between patients with and without complications. Mortality rates (%) were compared with the chi-square test, hospital length of stay (mean ± SD) and costs (mean ± SD) were compared with the two-sample *t*-test with unequal variance.

## Results

**Figure 1:**
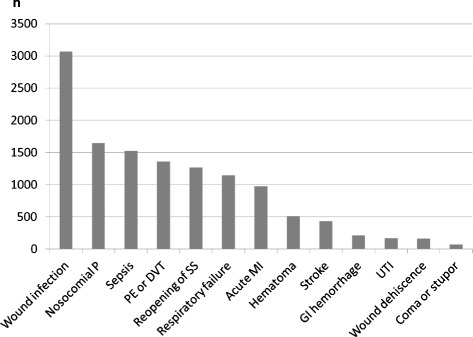
**Type (x axis) and number (y axis) of postoperative complications queried in the study population (75,140 patients).** P, pneumonia; SS, surgical site; PE, pulmonary embolism; DVT, deep venous thrombosis; MI, myocardial infarction; GI, gastro-intestinal; UTI, catheter-associated urinary tract infection.

A total of 75,140 patients from 222 medical centers met the search criteria. Numbers of patients per surgery group are reported in Table 2. 8,421 patients developed one or more of the 13 postsurgical complications extracted from the database (morbidity rate 11.2%). The most common postsurgical complication was wound infection (4.0%), followed by sepsis (1.8%), nosocomial pneumonia (1.7%), reopening of surgical site (1.6%), pulmonary embolism or deep venous thrombosis (1.5%), and respiratory failure (1.4%). All other complication rates were less than 1% (Figure 1). Morbidity rates for each surgery group are presented in Table 2.

**Table 2:** **Clinical and economic characteristics of the study population**

**Surgery**	**Patients, n**	**Morbidity, %**	**Mortality, %**		**HLOS, days**		**Direct cost, $**	
					**(mean ± SD)**		**(mean ± SD)**	
			**With**	**Without**	**With**	**Without**	**With**	**Without**
**AAA open repair**	2,040	19.6	20.8	6.0	23.9 ± 17.2	10.3 ± 8.2	76,169 ± 55,530	30,451 ± 24,023
**Vascular bypass**	6,765	9.5	10.3	1.1	17.5 ± 15.2	7.3 ± 6.5	42,202 ± 39,618	16,790 ± 12,601
**Esophagectomy**	1,794	12.5	6.3	2.4	23.2 ± 15.5	13.1 ± 11.7	59,382 ± 48,850	32,457 ± 30,571
**Gastrectomy**	5,995	8.7	11.7	1.0	25.0 ± 21.9	6.8 ± 7.7	54,879 ± 45,868	16,159 ± 15,986
**Colectomy**	19,055	16.0	15.2	2.6	23.1 ± 25.2	9.6 ± 8.2	49,160 ± 56,975	17,158 ± 16,481
**Resection of rectum**	4,251	9.2	4.9	0.4	16.2 ± 13.0	7.1 ± 5.3	29,874 ± 27,882	13,723 ± 10,020
**Hepatectomy**	4,934	7.6	14.8	0.7	17.9 ± 16.6	6.3 ± 4.4	48,961 ± 50,382	16,501 ± 12,080
**Pancreatectomy**	6,564	14.6	11.4	0.4	21.7 ± 19.1	9.7 ± 6.9	53,217 ± 50,882	20,888 ± 15,390
**Cystectomy**	4,036	10.9	5.2	0.4	19.3 ± 13.0	9.1 ± 5.4	43,598 ± 34,224	20,669 ± 10,511
**F&H fracture repair**	19,706	7.3	10.6	0.9	14.6 ± 12.2	6.6 ± 5.9	33,890 ± 33,115	14,919 ± 13,575

In-hospital mortality, hospital length of stay (HLOS) and direct costs were compared between patients with one or more complications (With) and patients without any complication (Without). All comparisons were statistically significant with a *P*-value <0.001. AAA, abdominal aortic aneurysm; F&H, femur and hip.

### Clinical impact of postsurgical complications

In patients with one or more complications and in patients without any complication in-hospital mortality was 12.4% and 1.4% (difference 11.0%, *P* <0.001), and mean hospital length of stay was 20.5 ± 20.1 and 8.1 ± 7.1 days (difference 12.4 days, *P* <0.001), respectively. The impact of postsurgical complications on in-hospital mortality and hospital length of stay for each surgery group is presented in Table 2.

### Economic impact of postsurgical complications

Average direct cost was $47,284 ± 49,170 and $17,408 ± 15,612 (difference $29,876, *P* <0.001) per patient with one or more complications and per patient with no complications, respectively. Thus, in 2011 the UHC spent a total of $252 M ($29,876 × 8,421 patients) to treat postsurgical complications in the study population. The economic impact of postsurgical complications for each surgery group is presented in Table 2.

### Projected cost-savings with implementation of GDFT

**Figure 2:**
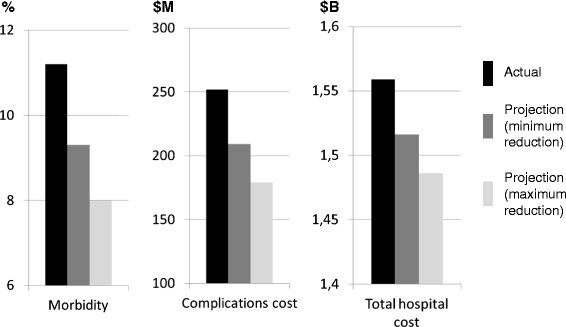
**Actual and projected morbidity rates, complication costs and total hospital costs with goal-directed fluid therapy implementation.**

**Figure 3:**
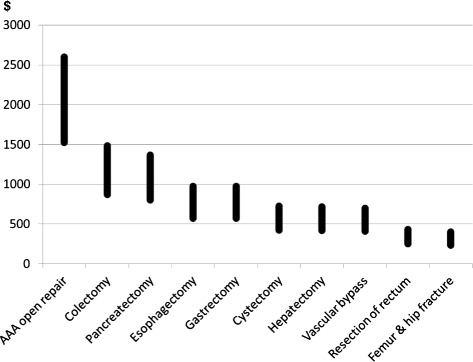
**Projected cost-savings per patient with perioperative goal-directed fluid therapy.** Each vertical black bar represents the range between minimum and maximum savings. AAA, abdominal aortic aneurysm.

**Figure 4:**
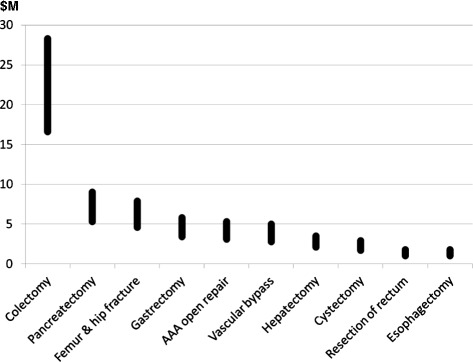
**Projected total cost-savings for the University HospitalSystem Consortium study population with perioperative goal directed fluid therapy.** Each vertical black bar represents the range between minimum and maximum savings.

The projected number of patients developing one or more complication, assuming an odds ratio ranging between 0.71 and 0.83, was 5,979 to 6,989 (morbidity rate 8.0 to 9.3%, Figure 2). Thus, after implementation of GDFT, projected gross savings would be $569 to $970 per patient and $43 to 73 M for the entire UHC study population (Figure 2). Projected cost savings for each surgery group are presented in Figures 3 and 4.

## Discussion

In our large UHC patient population, the occurrence of one or more postsurgical complications had a dramatic impact on in-hospital mortality (multiplied by 9.0) and hospital length of stay (multiplied by 2.5). Although impressive, these findings are consistent with the results of a recent study [30] done in 34,256 surgical patients discharged from a nonprofit 12-hospital system in the southern US. In that study, in-hospital mortality was 21-fold greater (12.3 versus 0.6%) and hospital length of stay was 4.7-fold greater in patients who developed one or more postsurgical complications.

Postsurgical complications also had a very significant impact on costs (+172%), the extra costs for treating patients developing one or more complications exceeding by far the cost of the surgical procedure itself ($29,876 versus $17,408). Our cost findings are consistent with previous and smaller studies. In 1,200 patients undergoing major abdominal surgery, Vonlanthen *et al*. [31] showed that patients with an uneventful course had mean costs per case of $27,946, whereas patients with one or more complications had a mean cost per case $34,446 higher. In 2,250 patients undergoing general and vascular surgery, Boltz *et al*. [32] showed that for patients developing one, two, and three or more complications the excess costs were $6,358, $12,802 and $42,790, respectively. In the above mentioned 34,256-patient cohort study [30], including major and minor procedures (for example, appendectomy), the average cost difference between a patient with and without complications was $22,398. These findings demonstrate the dramatic impact of complications on hospital costs and highlight a relevant savings capacity for major surgical procedures.

In our study, potential cost savings related to the use of GDFT were substantial, with projected gross savings of $569 to $970 per patient treated. This analysis revealed that potential savings per patient depend on the surgical procedure, ranging from $235 to $402 for femur and hip fracture to $1,523 to $2,599 for abdominal aortic aneurysm open surgery (Figure 3). Given the volume of procedures, total savings for UHC were the most significant ($16.6 M to 28.3 M) for colectomy (Figure 4). Our cost-saving estimations are lower than those reported by a previous UK prospective trial [13] showing a cost reduction of £1259 ($1889) per patient, and much lower than another trial [12] reporting £3467 ($5201) cost-savings per patient when GDFT was used. As far as we know, these are the only two prospective studies thus far comparing the cost for treating surgical patients with and without GDFT. These single-center evaluations have been done in a limited number of patients monitored with a pulmonary artery catheter 14 and 20 years ago in UK hospitals. Anesthesia and surgical practices, as well as healthcare costs, have dramatically changed over the last decades, rendering their applicability to current practices in the US questionable. We provide in this article a cost-saving estimation based on the most recent GDFT meta-analysis and a very large number of patients. This is the first clinical and economic prediction of its kind based on real US data. Our estimations are also lower than a recent cost simulation done in Sweden and suggesting that €1882/patient ($2258) could be saved if GDFT was to become the new standard of care in elderly patients with hip fracture [33].

Although free solutions have been proposed for GDFT [34], cardiac output monitoring techniques such as transesophageal Doppler and arterial-pulse contour methods are often used [35]. The cost of these technologies varies a lot from one region to the other (depending on reimbursement policies) and from one hospital to the other (depending on the volume of products used or bought every year by hospitals or group purchasers, respectively). If one assumes the cost of GDFT technologies, including capital investment for hemodynamic monitors and disposable sensors, to be approximately $300/patient in the US, the net savings are projected to be $269 to $670 per treated patient. When considering the implementation of GDFT, potential additional costs related to staff training and change in fluid or/and drug usage may also be considered. Training and technical support are usually provided (at least in part) by cardiac output monitoring companies, the use of vasoactive and inotropic drugs is not part of most recent GDFT guidelines [6-8], and recent clinical studies have shown that the net effect of GDFT strategies is usually no change [5] if not a decrease [36] in the total amount of fluid administered to patients during the perioperative period. However, GDFT costs may significantly vary from one medical center to the other, depending on the hemodynamic parameter used for GDFT (pulse pressure variation versus stroke volume), the cardiac output monitor amortization (large volume versus low volume of surgery), and the fluid used (albumin versus crystalloid). Therefore, another way to look at our data is to consider the potential savings of $569 to $970/patient as the upper limit for all GDFT-related costs. Then, a fair evaluation of the potential savings can be made on a case by case basis.

Our analysis probably underestimates the potential scope of the financial benefit of GDFT for several reasons. First, the analysis was strictly limited to major surgery in which outcome has already conclusively been shown to be improved by the use of GDFT. There are other types of surgery, such as major orthopedic spine, solid organ transplantation, and major gynecologic surgery, in which this approach would likely be associated with fewer complications [6,19]. Second, the UHC database, being an administrative database, has the advantage of containing reliable financial information, but also the disadvantage of underestimating the real incidence of postsurgical complications. In a comparison study, Steinberg *et al*. [37] reported a 28% morbidity rate with the National Surgical Quality Improvement Program (NSQIP) database (a clinical database specifically designed to collect up to 21 complications) and an 11% morbidity rate with the UHC database in the same surgical population. In the same study [37], the incidence of wound infection was 13% and only 1% with the NSQIP and the UHC database, respectively. Third, we used the odds ratio reported by the most recent GDFT meta-analysis [5] to estimate the potential reduction of postoperative morbidity. Because quality of care has improved over time, results of meta-analysis may be questioned because they include studies done a long time ago. A meta-analysis published in 2011 [4] looked specifically at the effects of GDFT over time. Interestingly, the morbidity reduction with GDFT was observed similarly for studies published in the 1980s, the 1990s and after 2000. The odds ratio for studies published after 2000 was 0.38 (0.29 to 0.50). If we had used this odd ratio, the projected savings would have been almost two times greater than those we report in the present study. Finally, our estimation does not take into account costs related to hospital readmissions. Postsurgical complications are the main cause for hospital readmission and it has been recently suggested that reducing complications may be the most efficient way to decrease readmissions and related costs [38].

Our analysis was limited to academic centers. The savings per patient may be different when the private sector is considered, and the total potential savings to the US healthcare system would be considerably more if the private sector was to be included. The potential clinical benefits and hence cost savings were derived from a meta-analysis, which could have incorporated reporting bias, with positive studies more likely to have been published. We considered the same postsurgical morbidity reduction with GDFT for all surgical procedures. Although previous meta-analyses [4,5] did not find any interaction between the type of surgery and the effect of GDFT, this may not always be true. Also patients with co-morbidities may benefit more from GDFT, but this is not something we were able to study. In addition, the assumption was made that there was no GDFT being used in 2011, whereas a survey published the same year indicates that approximately 5% of US anesthesiologists consistently use GDFT for high-risk surgery [9]. Further, this study assumes complete implementation of GDFT, which may be an unrealistic goal. The study by Kuper *et al*. [19] is the only real-life and large-scale GDFT implementation experience we know. They reported an adoption rate of 65%. Assuming a comparable adoption rate, our projected savings would have ranged between $370 and $631 per patient. Finally, if costs and savings are of utmost importance for payers, profits (reimbursement – cost) are even more important for hospitals [30]. We did not have access to reimbursement information so that we were unable to project the impact of GDFT on hospital profits. Prospective studies are definitely required to assess the impact of GDFT implementation not only on costs but also on hospital profits since this may become the main driver for hospital adoption.

## Conclusion

Our study demonstrates the dramatic impact of postsurgical complications on costs (+172%) in patients undergoing major surgical procedures at UHC hospitals, and suggests significant savings if GDFT was to be implemented. Projected cost-savings per patient are the highest for open repair of abdominal aortic aneurysm. However, taking into account the volume of surgery, the highest total savings for the UHC are expected to come from the implementation of GDFT in colectomy. Outside the UHC system, cost-savings are necessarily institution-specific, depending upon local case mix, morbidity rates, surgical costs and GDFT-related costs. Individual institutions can use our methodology, on a local level, in their decision to pilot and implement GDFT. Finally, we believe this analysis provides the necessary data to warrant a large and prospective study on the economic impact of GDFT.

## Key messages

- We assessed the costs of postsurgical complications in 75,140 patients undergoing 10 major abdominal, orthopedic, vascular and urologic surgical procedures in order to predict potential savings with perioperative goal-directed fluid therapy (GDFT)
- Postsurgical complications were responsible for a 172% increase in hospital costs (from $17,408 to $47,284 per patient)
- Projected cost-savings with the implementation of perioperative GDFT ranged between $569 and $970 per patient
- Highest cost-savings per patient ($1,523 to $2,599) were expected to come from the implementation of GDFT for open repair of abdominal aortic aneurysm.
- Given the volume of surgery, highest savings for the entire study population ($16.6 M to 28.3 M) were expected to come from the implementation of GDFT in patients undergoing colectomy

## Abbreviations

GDFT: goal-directed fluid therapy
UHC: University HealthSystem Consortium
